# The Role of miR-23b in Cancer and Autoimmune Disease

**DOI:** 10.1155/2021/6473038

**Published:** 2021-11-03

**Authors:** Yu-Xin Guo, Na Wang, Wen-Cheng Wu, Cui-Qin Li, Rui-Heng Chen, Yuan Zhang, Xing Li

**Affiliations:** ^1^National Engineering Laboratory for Resource Development of Endangered Crude Drugs in Northwest China, Key Laboratory of Medicinal Resources and Natural Pharmaceutical Chemistry (Shaanxi Normal University), The Ministry of Education, College of Life Sciences, Shaanxi Normal University, Xi'an, Shaanxi 710119, China; ^2^Surgical Oncology Department, The First People's Hospital of Tianshui, Tianshui, Gansu 741000, China; ^3^The High School Affiliated to Shaanxi Normal University, Xi'an, Shaanxi 710119, China

## Abstract

Short-stranded miRNAs are single-stranded RNA molecules involved in the regulation of gene expression. miRNAs are involved in a variety of cellular physiological processes, including cell proliferation, differentiation, and apoptosis. miR-23b have been identified to act both as oncogenes and as tumor suppressors. In addition, miR-23b is related to inflammation resistance to various autoimmune diseases and restrained inflammatory cell migration. The characterization of the specific alterations in the patterns of miR-23b expression in cancer and autoimmune disease has great potential for identifying biomarkers for early disease diagnosis, as well as for potential therapeutic intervention in various diseases. In this review, we summarize the ever-expanding role of miR-23b and its target genes in different models and offer insight into how this multifunctional miRNA modulates tumor cell proliferation and apoptosis or inflammatory cell activation, differentiation, and migration.

## 1. Introduction

According to GLOBOCAN 2020, an assessment of cancer morbidity and mortality, it is reported that the number of new cancer cases reached 19.3 million worldwide, and almost 10 million people died from cancer [1]. Moreover, breast cancer in women has overtaken lung cancer as the primary cause of cancer incidence worldwide in 2020 [1, 2]. Then, lung cancer is the second most frequently occurring cancer and the leading cause of cancer death [1]. Moreover, changes in incidence and trends are closely related to the prevalence of tobacco [3, 4]. So, men are more likely to suffer from this disease. Among male cancers, liver cancer is also a high incidence disease, ranking second in male mortality, and the incidence of primary liver cancer has continued to rise since 2020 [4, 5]. Gastric cancer is a significant disease worldwide. Notably, in the United States, Canada, and the United Kingdom, the incidence of gastric cancer has increased in both low- and high-risk young adults (younger than 50 years) [6]. At present, the treatment of tumors can be divided into drug therapy and surgical treatment [7]. Drug therapy refers to using drugs to destroy cancer cells, which is often used in clinical treatment. However, while killing tumor cells, it will kill normal cells, so it often brings a series of side effects, and chemotherapy does not have specificity for tumor tissue [8]. Thus, most drug therapy has side effects. In addition, surgical treatment has adverse effects such as postoperative recurrence and slow healing. Importantly, their pathogenesis is also unclear [9]. These factors lead to limited treatment options. Therefore, clarifying the specific mechanism of the disease is of great significance for the treatment of the disease.

Autoimmune disease refers to a disease in which the body's immune response to its antigen causes damage to its own tissues [10]. Multiple sclerosis (MS) is an autoimmune demyelinating central nervous system (CNS) disease, in which immune cells infiltrate into the central nervous system from the periphery, activate microglia and astrocytes, and inhibit the differentiation of oligodendrocytes into oligodendrocytes, resulting in pathological features such as demyelination of myelin and axon [11, 12]. However, its exact molecular mechanisms remain unclear. Besides, rheumatoid arthritis (RA), also a chronic autoimmune disease, affects nearly 0.5%–1% of the population in the world [13]. The most common clinicopathological features of RA patients are cartilage degeneration and bone erosion of large and small joints, leading to mobility difficulties and even disability in severe cases [14, 15]. Although there is some genetic and environmental correlation, the specific pathogenesis is not clear [16]. Systemic lupus erythematosus (SLE) is also a chronic multisystem autoimmune disorder. Although the cause of SLE is unknown, both genetic and environmental elements are relevant to the disease mechanism [17]. Infection and environmental elements have been hypothesized to cause cell damage, promote the exposure of autoantigens to the immune system, and cause B- and T-cell activation [18]. Indeed, clarifying the pathogenesis plays a critical role in the diagnosis and timely treatment of diseases.

Small endogenous regulatory RNAs, also known as short-strand ribonucleic acid microRNAs (miRNAs), are critical posttranscriptional regulators of gene expression and were first identified in *C. elegans* [19–21]. There are many kinds of miRNAs, among which miRNA-23b belongs to miR-23b/27b/24-1 cluster [22]. miR-23b possessed regulatory roles, especially in the development of cancer and autonomic immune diseases [23]. In conclusion, this review reveals miR-23b in various diseases, including cancer and autoimmune diseases, and its role in disease progression.

## 2. miRNA

MicroRNA (miRNAs), which belonged to a category of single-stranded RNA molecular, is not involved in coding with a role in regulating gene expression [24, 25]. The formation of miRNA includes the multistage process. Firstly, in the nucleus, RNA polymerase II or III transcripts miRNA-related genes into primary miRNAs (pri-miRNAs), where miRNAs are several thousand nucleotides (nt) long [26–28]. Subsequently, the microprocessing complex Drosha-DGCR8, consisting of the RNA binding protein DiGeorge syndrome critical region gene8 (DGCR8) and the ribonuclease type III RNase Drosha, splits the precursor miRNA (pre-miRNA), which forms the hairpin structure [29, 30]. This process is carried out in the nucleus. Then, in the cytoplasm, the RNA Dicer enzyme decomposed per-miRNA into mature miRNA, and the miRNA was still in the double-stranded state [29]. Finally, the double-stranded miRNA combined with Argonaute2 (AGO2) to form RISC (RNA-induced silencing complex) [31]. One strand of the miRNA double strand is preserved in the RISC complex, while the other strand is expelled from the complex and rapidly degrades [30]. In the cytoplasm, miRNAs exert various biological functions by RISC [32]. miRNAs processing and loading into RISC is performed by specific RNA-biding proteins (RBPs), which exert cotranscriptional and posttranscriptional regulation of miRNA transcription product [33]. Moreover, a number of miRNAs can have different nuclear functions independently of RISC [33].

MicroRNA regulation commonly occurs based on microRNA binding to the 3′ untranslated region (3′-UTR) of target mRNA [34]. MicroRNAs inhibit the expression of target genes by 3′-UTR combining with target RNAs [35]. Therefore, different miRNA biological processes occur at different sites in the cell, including RNA transcription, processing, transport, and RISC binding. Importantly, miRNAs are critical for cell proliferation, differentiation, and apoptosis [36]. miRNAs have been involved in many cancers and neurodegenerative diseases, such as multiple sclerosis, Parkinson's disease, and Alzheimer's disease [37, 38]. Overall, miRNAs play an essential part in the occurrence and development of diseases [39].

## 3. miR-23b Research Progress

The miR-23b is due to the chromosomal region 9q22,32 encoding mi-23b/27b/24-1 [23]. The biogenetic process of miR-23b is similar when miRNA is cut into miRNA double strand by Dicer enzyme-containing protein complex. One strand is a passenger strand that will be degraded, and the other is miR-23b. miR-23b is involved in regulating normal physiological function, cell differentiation, and cellular immunity [40]. Thus, when the miR-23b homeostasis is damaged, the normal physiological function of the cell will also be affected, and then diseases will occur. miR-23b can induce a complex network of responses by directly targeting multiple transcripts. To be specific, the changes of miR-23b expression were closely related to various transcription factors, such as TAB2, TAB3, NF-*κ*B, tumor suppressor P53, estrogen receptor ER-*α*, mitogen-activated protein kinase MAPK, activation protein AP-1, reactive oxygen species ROS, and CCL7 [19, 41, 42]. It has been reported that miR-23b is closely related to the occurrence and development of a variety of diseases, including tumors and autoimmune diseases [23]. This review summarized tumor-related diseases such as breast cancer, lung cancer, gastric cancer, and liver cancer [43–45] and autoimmune diseases such as multiple sclerosis, systemic lupus erythematosus, and arthritis [46–48]. The above studies indicated that miR-23b is mainly involved in a variety of physiological processes such as cell proliferation, migration, and adhesion [49, 50].

## 4. The Role of miR-23b in Cancer

### 4.1. Breast Cancer

miR-23b is a pathogenic gene in the course of the occurrence of breast tumors. Because miR-23b expression changes abnormally in breast cancer, it is considered a biomarker for breast cancer development. The expression rate of miR-23b in breast cancer tissues was significantly higher than that in benign breast fibroadenomas. Through KEGG pathway enriching analysis, it is found that miR-23b is involved in the metabolism and cellular pathway of breast cancer, such as EGFR and c-Met signaling pathways [51, 52]. In addition, the function of miR-23b at the cellular and molecular level has also been extensively studied. The CRISPR/Cas9 system was able to knock out miR-23b and miR-27b thoroughly; therefore, some researchers used this system to knock out the miR-23b gene in MCF-7 cells. The results showed that the cell behaviors were changed, such as cell growth rate and colony formation, and significantly decreased [53, 54]. Moreover, miR-23b expression is regulated by multiple factors. A study shows that the membrane receptor tyrosine kinase (HER2/neu) can induce miR-23b by regulating its downstream transcription factor NF-ᶄB, promoting the growth of breast cancer cells [55]. On the other hand, miR-23b blockades tumor cell invasion by inhibiting the expression of B-lymphocyte-induced maturation protein-1 (Blimp1) [43, 56]. Cas/ErbB2 MCF10A.B2 represents invasive human mammary epithelial cells with characteristics of overexpression p130Cas and activation of ErbB2. miR-23b can directly reversely mediate Blimp1 and increase its level of expression [43].

### 4.2. Lung Cancer

miR-23b is identified to be related to lung cancer according to a variety of validation methods, including PCR array, logistic regression, and receiver operating characteristics curve analyses; mir-23b is determined to be closely related to the formation of lung cancer [57]. By performing an MTT assay, it was demonstrated that, in the H1838 lung cancer cell line, the overexpression of miR-23b significantly improved cell viability. In H1437 and H1944 lung cancer cell lines, inhibiting the expression of miR-23b significantly reduced the ability of cell proliferation [45]. The specific mechanisms of action indicate that, by increasing the expression of miR-23b, it acts on myeloid leukemia 1 short (Mcl-1S) gene to enhance the proliferation, migration, and invasion ability of A549 cells [58]. Mcl-1S has a proapoptosis effect, which is a short splicing variant of antiapoptosis protein Mcl-1 [59]. This may be the main reason that miR-23b can promote the growth of lung cancer cells. A new study proved that kinectin1 antisense RNA 1 (KTN1-AS1) is negatively correlated with miR-23b in NSCLC (non-small-cell lung cancer) cells, and the overexpression of KTN1-AS1 can significantly reduce the expression level of miR-23b. Administration of KTN1-AS1 can restore the proliferation and growth of NSCLC cells [60]. KTN1-AS1 contributes to facilitating NSCLC progression by inhibiting miR-23b [60].

### 4.3. Liver Cancer

There is a critical relationship between liver cancer and immunity [61, 62]. The liver acts as an immune organ, maintaining immune homeostasis and containing many immune cells, such as DC cells and T cells [61]. Tregs are an immunosuppressive subset of CD4+ T cells. Tregs have an important feature; that is, they have both activating and inhibitory receptors. Blocking activated receptors and/or stimulating inhibitory receptors shifts the balance to inhibiting Tregs, treating tumors and chronic infectious diseases. Furthermore, Tregs play a crucial role during tumor development and progression by regulating other immune cells. Notably, Tregs work with neutrophils to reduce the incidence of liver cancer [63]. On the contrary, the therapeutic effects of Treg can be achieved by blocking the inhibitory receptors or stimulating the activation receptors in autoimmune diseases [62]. According to the reports, autoimmune liver disease is related to the number and functional defects of Tregs. Therefore, the treatment of autoimmune liver disease aims to restore the sufficient number and function of Treg [64, 65].

In hepatocellular carcinoma (HCC) cells, miR-23b possessed important functions [66]. miR-23b may possess a dual function of oncogenic and inhibitory effect on the tumor. Because the expression of miR-23b is detected in 125 HCC patients, 48 of them were upregulated, and 77 were downregulated [67]. In Cao's research, it was shown that the expression of miR-23b in HCC tissues was remarkably decreased, which was positively correlated with metastasis of HCC [44]. Intriguingly, body fat is also associated with the progression of liver cancer. Compared with HCC patients with low body fat percentage, the study has found that serum exosomes of HCC patients with a high body fat ratio express a high level of miR-23b [68]. Besides, hepatocellular carcinoma cell line SMMC-7721 demonstrated that miR-23b could promote tumor cell growth by targeting suppression of tumorigenicity 7 like (ST7L) [66]. Proline-rich tyrosine kinase 2 (PYK2) is a nonreceptor tyrosine kinase belonging to the adhesion-focused kinase family [69]. PYK2 plays an essential role in regulating cell proliferation and migration in various cancer cells [70, 71], and miR-23b inhibits the HCC cell line MHCC97L by targeting Pyk2 [72].

### 4.4. Gastric Cancer

The high expression of miR-23b, a typical feature of gastric cancer, is believed to facilitate this disease's aggressive progression [73]. Moreover, miR-23b in plasma expression is correlated with a poor prognosis of gastric cancer [74]. miR-23b is one of the critical factors in the initiation and progression of gastric cancer. By performing experiments in a gastric cancer xenograft mouse model and gastric cancer cells MKN-45 and AGS, results can identify that miR-23b could target programmed cell death (PDCD4) and promote tumor growth [75]. Besides, it has demonstrated that miR-23b and long noncoding RNA, tumor suppressor candidate 7 (TUSC7), inhibited each other. Contrary to the effect of miR-23b, TUSC7 suppressed the growth of gastric cancer cells AGS and MKN-45 [76]. The latest clinical data has shown that miR-23b encapsulated in the exosomes can also be used as a biomarker to predict the recurrence and prognosis of gastric cancer patients at different stages [77].

**Figure 1:**
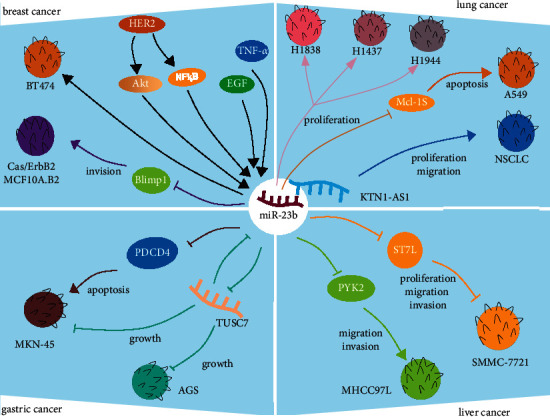
The mechanism of miR-23b in cancers. (1) Breast cancer: HER2, EGF, and TNF-*α* promote the growth of BT474 cells by promoting the upregulation of miR-23b. Cas/ErbB2 MCF10A.B2 represents overexpression p130Cas with activation of ErbB2. miR-23b impairs Cas/ErbB2 MCF10A.B2 cell invasion by downmodulating Blimp1 expression. (2) Lung cancer: miR-23b promotes H1838, H1437, and H1944 lung cancer cell proliferation. It is beneficial for the growth of A549 by Mcl-1S. In addition, KTN1-AS1 promotes NSCLC proliferation by inhibiting miR-23b. (3) Liver cancer: miR-23b boosts the proliferation of H1838, H1437, and H1944 lung cancer cell lines. It is useful for the expansion of A549 by Mcl-1S. Furthermore, KTN1-AS1 accelerates NSCLC proliferation by inhibiting miR-23b. (4) Gastric cancer: miR-23b modulates tumor growth by targeting PDCD4. Moreover, as a potential target of miR-23b, TUSC7 also regulates the growth of gastric cancer cells AGS and MKN-45.

The mechanism of miR-23b in breast cancer, lung cancer, liver cancer, and gastric cancer is shown in Figure 1.

## 5. The Role of miR-23b in Autoimmune Disease

### 5.1. MS/EAE

Abnormal expression of a series of microRNAs can be used as potential therapeutic targets for EAE, assessed in the plasma and spinal cord tissue of EAE mice [78]. In addition to the dysregulation of miR-23b in the tumor diseases mentioned above, miR-23b also reflected abnormal expression in autoimmune diseases. The analysis of a miRNA-microarray found that, with the aggravation of EAE, the expression of miR-23b gradually increased. This result is considered to be one of the biomarkers of the disease [79]. Moreover, several studies have reported that miR-23b regulates autoimmune disease pathogenesis by targeting different protein molecules, such as TAB2, TAB3, IKK-*α*, and CCL-7.

Bone marrow mesenchymal stem cells (BMSCs), adult pluripotent stem cells, exert the immunoregulatory role by carrying miRNA. BMSCs combined with miR-23b had a better synergistic effect and could effectively alleviate EAE [46]. BMSC loading overexpression miR-23b inhibits Th17 cell differentiation, blocks the secretion of inflammatory factor IL-17, on the contrary promotes the secretion of tumor growth factor-beta 1 (TGF-*β*1), and ultimately inhibits the development of EAE [46]. In addition to analyzing the effect of miR-23b on EAE verification from the perspective of inflammatory subset cells Th17, the research focuses on the effect of inflammatory chemokine CCL7. Similar to the effect of miR-23b in Th17 cells, miR-23b inhibits Th1 and Th17 cells and diminishes the infiltration of encephalitogenic T cells into the central nervous system contributing to halting EAE by binding with CCL7 in the 3′-UTR site [41]. In addition, miR-23b could alleviate the severity of EAE by targeting TAB2, TAB3, and IKK-*α* [42].

### 5.2. RA

RA chronically damages the heart, skin, and many other organs, accompanied by pathological characteristics of erosive changes in joint surfaces that lead to the destruction of the joints [80]. Besides its specific expression in MS, miR-23b is also expressed explicitly in arthritis. It is therefore considered to be a biomarker of RA [47]. The identification of miR-23b expression shows downregulation in inflammatory lesions from RA individuals and related mouse models compared with healthy controls [42]. It is well known that RA is more common in old age [81]. However, juvenile idiopathic arthritis will also occur in a high proportion, which is very detrimental to the growth of children [82]. The study has shown that miR-23b helps in the diagnosis and monitoring of RA [83]. miR-23b is negatively related to inflammation in RA [47]. Similarly, the negative correlation between IL-17 and miR-23b is verified in comparing RA patients and healthy subjects [42]. In addition, Zhu et al. found that (TAB2), TAB3, and nuclear factor k-B kinase subunit α (IKK-α) were down-regulated after transfection of miR-23b in fibroblast-like synovial cells (FLSs), which were obtained from synovial joint tissues of individuals with knee joint injury. [42]. Therefore, it is implied that miR-23b can target TAB2, TAB3, and IKK-*α* to alleviate disease.

### 5.3. SLE

SLE is an autoimmune disease in women with features of multiple tissues and systems [84]. Moreover, brain tissue is often the target organ of this disease. Due to the long-term and widespread existence of intracranial vascular inflammation, part of the gray matter shows ischemia, infarction, atrophy, and several demyelinations of white matter areas [85]. The injury of the gray and white matter directly affects nerve function and leads to abnormal clinical symptoms. The incidence of SLE is increasing year by year, with facial erythema, joint pain, fever, and fatigue as the primary manifestations. Using the mouse model of SLE, the research has verified that the treatment of adipose-derived stem cells (ADSCs) can effectively alleviate the progression of the disease. Specifically, it reduces the expression of inflammatory factor IL-17, which may be related to the upregulation of miR-23b [48]. Additionally, through RNA differential analysis of renal biopsy samples from several patients with SLE, it is found that miR-23b is downregulated in the inflammatory sites of SLE patients [42]. Similar to RA, miR-23b also inhibited the development of SLE upon inhibiting TAB2, TAB3, and IKK-*α* [42]. All in all, the high expression of miR-23b will be helpful for the relief of SLE.

**Figure 2:**
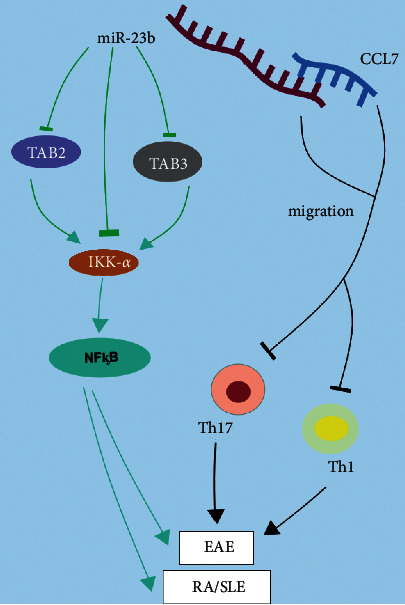
The mechanism of miR-23b in MS, RA, and SLE. IKK-*α* could promote the expression of inflammatory factor NF-ᶄB, which contributes to the occurrence of autoimmune diseases RA, SLE, and MS/EAE. Additionally, miR-23b could alleviate these diseases by inhibiting TAB2 and TAB3, which are beneficial for IKK-*α*. Besides, the binding of miR-23b to CCL7 can inhibit the migration of inflammatory cells Th1 and Th17 and ultimately inhibit disease development.

The mechanism of action of miR-23b in EAE, RA, and SLE is summarized in Figure 2.

## 6. Treatment

miRNAs are abnormally expressed in various pathological processes. Restoring miRNA normal levels might be regarded as a promising therapy. Up to date, miRNA inhibitors have been frequently used in the study of miRNA function and mechanism. It is common to use artificial inhibitors, including anti-miRNA oligonucleotide (AMO) and miRNA sponges [86]. AMO is a short-stranded RNA oligonucleotide that is complementary to natural miRNAs [87]. AMO has been used in various cancers [88–91]. Artificial miRNA sponge is constructed by inserting tandemly arrayed miRNA sites into 3′-terminus (3′ 3′-UTRs) of a reporter gene [92]. This type of miRNA sponge is characterized by inductive and stable expression, driven by the most potent promoters in mammalian systems, such as U6 or cytomegalovirus (CMV) [93]. MicroRNA (miRNA) sponges are transcripts with repeated miRNA antisense sequences that can sequester miRNAs from the endogenous target, leading to miRNA translation inhibition or mRNA degradation to fail [94]. The microRNA sponge can play a role in cancer treatment. For example, there have been studies based on the bladder cancer xenograft model of BALB/c nude mice, and lentivirus-transduced miR-130b/miR-494 sponge inhibits tumor growth [95]. Besides, the miRNA sponge also has excellent potential in the treatment of liver cancer. To be specific, miR-17-3p, miR-181b-5p, and miR-9 sponges all demonstrated the ability to inhibit the growth of liver cancer cells [96–98]. Interestingly, the team identified a circRNA that is highly expressed in human and mouse brains and later showed that this circRNA could act as a sponge for miRNAs [99, 100]. For example, circHIPK3 is the sponge of miR-558 inhibiting bladder cancer development both *in vivo* and *in vitro* [101]. Notably, miR-23b sponge is also applied to liver cancer cells and glioma cells, and the results show that it has a good effect in inhibiting the disease [102, 103]. In addition to the above methods for regulating miRNA levels, RNA mimics can be used as well. miRNA mimics are double-stranded RNA molecules, which modulate miRNA level [25]. miRNA mimic is a strategy to restore miRNA function. Even viral vectors can transfect miRNA into cells, but they have genome integration and the potential danger of immunogenicity [104]. For example, miR-125b-5p mimic has been demonstrated to inhibit acute liver injury in vivo [105]. Therefore, miRNA mimic does not integrate into the genome, making it a good prospect in disease treatment [104]. In conclusion, the AMO, sponge, and mimic of miR-23b can potentially treat cancers and autoimmune diseases.

## 7. Conclusion

miR-23b is frequently upregulated in a variety of tumors and human cancer cell lines and exerts a vital function in tumorigenesis. The expression level of miR-23b is induced by the HER2/neu, EGF, TNF-*α*, and Blimp1, constitutively activated in breast cancer [43, 55]. In studies on lung cancer cell lines and NSCLC cells, miR-23b has been shown to promote cancer development. Therefore, miR-23b is a potential clinical pathologic marker in lung cancer [57]. In addition, miR-23b can be used as a novel therapeutic target. Studies in liver cancer have shown that ST7L, as the direct target of miR-23b, plays a regulatory role in liver cancer cells and can act as an oncogene [66]. Finally, for gastric cancer, miR-23b promotes tumor development by targeting PDCD4 [75]. It has been shown to antagonize TUSC7, a tumor inhibitor [76]. However, the regulatory role of miR-23b looks paradox in different cancer. In conclusion, miRNA-23b expression profiles differ between disease states and normal tissue, and the abnormal regulation of miR-23b can be used as a warning for tumors in tumor studies. However, its regulatory effects on a variety of proteins make it a very challenging target for cancer therapy. In general, for tumors, miR-23b often has different roles in divergent systems or environments. It is consistent with previous research illustrating that one of the frustrating aspects of microRNA research is that individual microRNAs have opposite functions in different systems, suggesting that microRNA communication is environment-dependent [34]. Some examples demonstrate that miR-125b is downregulated in various cancers such as hepatocellular carcinoma and breast cancer and overexpressed in colon cancer and hepatocellular tumors [106]. Furthermore, future work should build on the study of how miR-23b participates in the tumor suppressor pathway or promotion pathway to lay a theoretical foundation for tumor therapy.

The discovery of miRNAs has expanded the knowledge of human diseases, including autoimmune diseases. Here, we have summarized the crucial functions of miR-23b as an anti-inflammatory gene in MS/EAE, RA, and SLE. Hundreds of cell- and animal-based studies agree on the inflammatory-suppressive role of miR-23b and suggest recovery of miR-23b level as a potential therapeutic approach. In autoimmune diseases, the overexpression of miR-23b primarily reflects the ability to inhibit the differentiation of th17 cells, reduce inflammatory cytokines, and block the infiltration of inflammatory cells into the lesion. The benefit of miR-23b-based therapy is the chance to suppress multiple proinflammatory cytokines and chemokines production concurrently in EAE [41, 42]. As a marker of RA, studies have shown that it can help diagnose and detect the disease. Moreover, it can downregulate Tab2, Tab3, and IKK-*α*. In SLE studies, studies in animal models have shown that overexpression of miR-23b can inhibit the inflammatory factor IL-17 and alleviate SLE. Next, miR-23b inhibitors can be administrated to observe whether they can consistently inhibit inflammatory factors secretion and disease development in animal models of SLE. Furthermore, a more detailed understanding of mechanisms underlying how miR-23b modulates therapeutic effect might be a study focus in the future.

Although miR-23b functions based on multiple pathways and multiple targets in the disease's pathological process, it is inevitable that the expression of miR-23b is abnormal and undulatory during the occurrence of the disease. Regulating miR-23b to its normal level is a new potential therapeutic strategy for treating related diseases. So far, the application of miRNA sponges, AMOS, and mimics has provided favorable conditions for regulating abnormal miR-23b expression.
